# CORRELATES OF LONELINESS AMONG OLDER ASIAN IMMIGRANTS: A SYSTEMATIC REVIEW

**DOI:** 10.1093/geroni/igz038.2345

**Published:** 2019-11-08

**Authors:** Yihan Wang, Bongki Woo, Nan Jiang

**Affiliations:** 1 Boston College, Boston, Massachusetts, United States; 2 University of Southern Carolina, Columbia, South Carolina, United States; 3 Columbia University, New York, United States

## Abstract

Loneliness is a prevalent social concern among older adults, which calls for attention as a condition itself and its influence on mental and physical conditions. However, limited efforts have been made to understand loneliness, particularly among immigrant older adults. Guided by the ecological perspective, the present study contributes to the literature by providing a systematic review of the prevalence and individual-, household-, and community-level correlates of loneliness among Asian older immigrants, one of the fast-growing immigrant population. Following the PRISMA guideline, we systematically searched eight electronic databases to identify relevant empirical research articles. Of the 828 articles identified, ten articles met the inclusion criteria. Majority of these articles focused on older Chinese and Korean immigrants. On the individual level, migration grief, longer length of residence, and weaker ethnic attachment were linked to higher level of loneliness, indicating that immigration can be a challenging experience for later life well-being of Asian older adults. Other identified correlates include mental and functional impairment and worsening health changes. On the household level, while living alone was a commonly identified correlate of loneliness, those who live with family also reported loneliness when they have fewer interactions with their family members. On the community-level, smaller social network and lack of social support and interactions were correlates of loneliness. The findings of the present study are helpful for identifying older Asian immigrants who may be at risk of loneliness and implicates that the efforts to mitigate the loneliness need to be made at various ecological levels.

